# A Meta-Analysis of Self-Reported Achievement Goals and Nonself-Report Performance across Three Achievement Domains (Work, Sports, and Education)

**DOI:** 10.1371/journal.pone.0093594

**Published:** 2014-04-03

**Authors:** Nico W. Van Yperen, Monica Blaga, Tom Postmes

**Affiliations:** Department of Psychology, University of Groningen, Groningen, The Netherlands; Tilburg University, Netherlands

## Abstract

During the past three decades, the achievement goal approach to achievement motivation has emerged as an influential area of research, and is dedicated to understanding the reasons behind the individual’s drive to achieve competence and performance. However, the current literature on achievement goals is segmented rather than integrated. That is, citations across the three major and distinct achievement domains (work, education, and sports) are more the exception than the rule and similarities and differences between findings for the different achievement domains have yet to be tested. The purpose of the present study was to examine the relationships between self-reported achievement goals and nonself-report performance through meta-analysis, and the moderating potential of achievement domain. Identifying achievement domain as moderator improves our understanding to which contexts we can (not) generalize conclusions to, it helps to understand seemingly inconsistent findings, and opens avenues for future research on the underlying processes. Because the achievement goal (AG) measure used in a study is partially confounded with achievement domain, we examined the moderating role of this variable as well. Our findings suggest that – overall – approach goals (either mastery or performance) were associated positively with performance attainment, whereas avoidance goals (either mastery or performance) were associated negatively with performance attainment. These relationships were moderated by achievement domain. For example, relative to the education or work domain, in the sports domain, we did not observe negative correlations between avoidance goals and performance. The absence of statistical moderation due to AG measure suggests that the observed moderation of achievement domain cannot be explained by the AG measure utilized. We suggest further steps to integrate the achievement goal literature, and accordingly, to broaden and deepen understanding of performance attainment in competence-relevant settings, including the workplace, the sports field, and the classroom.

## Introduction

The drive for performance is fundamental to human nature, and manifests itself across a variety of achievement domains, such as work (e.g., employees striving to do better than others in their work), sports (e.g., athletes aiming at performing better in their sports than they have done in the past), and education (e.g., students who want to get a lot of things right in their studies). The manner in which individuals define, experience, and respond to the specific competence-relevant situations that they encounter is partly a function of their achievement goals [1], [2]. During the past three decades, the achievement goal approach to achievement motivation has emerged as an influential area of research, and is dedicated to understanding the reasons behind the individual’s drive to achieve competence and performance [2]. However, the findings have been inconclusive due to divergence in the characteristics of the studies, tasks, and samples. Hence, the purpose of the present study was to systematically explore through meta-analysis the relationships between self-reported achievement goals and nonself-report performance, including the moderating potential of achievement domain. In addition, we explored the role of other possible moderators (achievement goal measure, age, sex, nationality, and publication status) that might explain the mixed and inconsistent findings. A meta-analysis is a quantitative summary of the pooled results of studies on the same topic, and thus provides more meaningful results than any individual study on its own [3], [4].

In previous reviews and meta-analyses [5]–[9], it was found that, in general, both mastery-approach goals (i.e., with a focus on doing better than one has done before) and performance-approach goals (i.e., with a focus on doing better than others) were related positively to performance attainment. In contrast, performance-avoidance goals (i.e., with a focus on not doing worse than others) and mastery-avoidance goals (i.e., with a focus on not doing worse than one had done before) were, in general, related negatively to performance attainment. However, in these reviews, *achievement domain* was largely overlooked as being a potential moderator of the relations between achievement goals and performance attainment. Either the focus was (almost) exclusively on the domain of educational psychology [6]–[8] or different achievement domains were combined into a single analysis [5], [9].

In the present study, we extended the scope of previous work by investigating the moderating potential of achievement domain (education, work, and sports). This is an important issue, because the current literature on achievement goals is segmented rather than integrated. That is, citations across achievement domains are more the exception than the rule [10], and similarities and differences between findings for different achievement domains have yet to be tested. Furthermore, identifying achievement domain as moderator improves our understanding to which contexts we can (not) generalize conclusions to, helps to understand seemingly inconsistent findings, and opens avenues for future research on the processes that underlie the possible different positive and negative effects of the different achievement goals on performance.

In our meta-analysis, we drew upon the 2×2 framework for achievement goals that was developed by Elliot [11], [12], in which goals are separated by definition (mastery versus performance) and valence (approach versus avoidance). In addition, we explored the moderating role of achievement goal (AG) measure, a variable that is partially confounded with achievement domain (which will be discussed below), as well as age, sex, nationality, and publication status.

### The Achievement Goal Approach to Achievement Motivation

The achievement goal approach to achievement motivation defines achievement goals as mental representations of the individual’s desired level of competence or undesired level of incompetence [2]. Initially, achievement goal theorists distinguished two types of achievement goal: mastery goals and performance goals [13], terms we use as labels throughout this article. Note that across various achievement domains, mastery goals have also been called “task” goals [47], [103] or “learning” goals [1], [45]. Performance goals have also been called “ability” goals in education [100], “prove” goals in work [15], and “ego” goals in sport [47], [50].

Individuals with mastery goals focus on self-referenced or task-referenced standards of competence. They define competence according to their personal improvement or mastery of the task. In contrast, individuals with performance goals focus on other-referenced standards of competence. They define competence according to how well they perform relative to others [2]. Initially, both mastery goals and performance goals were considered implicitly to be *approach* goals (but were not necessarily operationalized as such). This means that they were presumed to direct the individual towards attaining positive outcomes and desirable events, that is, improvement and development (mastery goals) versus outperforming other individuals (performance goals). Contradictory findings led to the addition of an *avoidance* component for both mastery and performance goals [11], [12], [14]–[16]. Hence, in contemporary research on achievement goals, achievement goals differ in terms of the standards that individuals use to define competence, i.e., a self-referenced or task-referenced standard (mastery) versus an other-referenced standard (performance), and valence (i.e., approach versus avoidance). Individuals who pursue mastery-approach (MAp) goals focus on task-referenced or self-referenced improvement and accomplishments, whereas individuals who pursue performance-approach (PAp) goals focus on performing better than others. Individuals who pursue mastery-avoidance (MAv) goals aim to avoid incompetence on the basis of task-referenced or self-referenced standards, whereas individuals who pursue performance-avoidance (PAv) goals focus on avoiding failure relative to others [2], [12], [17], [18].

### The Present Study

In the extant research on achievement goals, meaningful links between self-reported achievement goals and performance attainment have been documented using a variety of samples, which range from primary school children [19] to undergraduates [20]–[22], and from working adults [23], [24] to professional athletes [25]. The performance level of an individual is arguably a key outcome variable because it reveals valuable information about his or her potential to adapt to the achievement situation [26].

However, the relations between achievement goals and performance attainment seem to be rather inconsistent across studies. For example, MAp goals were found to be linked positively to performance across a variety of samples [23], [24], [27]–[34], but were also sometimes found to be unrelated to performance [16], [21], [22], [35]–[38]. PAp goals were found to be related positively to sports performance [39], [40] whereas in other studies, PAp goals were found to be unrelated to academic performance [41] and work performance [32]. Similarly, avoidance goals were related more negatively to performance in some samples [42] than in others [37]. These mixed and sometimes inconsistent findings might be explained by specific moderating variables, including achievement domain.

### Achievement Domain as Moderator

Although the literature on achievement goals spans three major and distinct achievement domains (work, education, and sports), to date the potential moderating role of achievement domain has been essentially ignored. Hence, we do not know to what extent findings in one domain are different from, or can be generalized to, other domains. Previous meta-analyses [6] and review articles [8], [41], [43], [44] focused typically on the domain of educational psychology. The few reviews that included more than one achievement domain collapsed all studies into one analysis [5], [9], or lacked the power to identify a possible moderating effect of achievement domain [7]. Across the domains of education, work, and sports, the social domain (e.g., interpersonal relationships with friends or peers), and “other” domains (e.g., computer games), Hulleman et al. [7] found that, in general, both MAp goals (*r = *.11) and PAp goals (*r = *.06) were related positively to performance, whereas PAv goals (*r* = −.13) and MAv goals (*r* = −.12) were related negatively to performance. In another recent meta-analysis, Baranik et al. [5] collapsed studies from the domains of work, education, and sports, and reported findings similar to those of Hulleman et al. [7]; namely, that performance attainment was related positively to both MAp goals (*r = *.10) and PAp goals (*r = *.13), and related negatively to both PAv goals (*r* = −.18) and MAv goals (*r = *−.09). Payne et al. [9] excluded samples of children and adolescents (which predominate in the domain of education), as well as studies from the domain of sports and, accordingly, focused on adults in educational and occupational settings. They reported links between achievement goals and two separate performance measures: academic performance and job performance. They found positive relations between MAp goals and academic performance (*r = *.16), and no relations between either PAp goals (*r = *.02) or PAv goals (*r* = −.06) and academic performance. With regard to job performance, the overall correlations were positive for both MAp goals (*r = *.18) and PAp goals (*r = *.11). No studies in their sample examined the link between PAv goals and job performance. Although Payne et al. [9] separated the two performance outcomes (academic performance and job performance), they did not test or discuss their findings as being a function of achievement domain (education versus work). In sum, the overall pattern of results across these previous meta-analyses on the link between self-reported achievement goals and (self-report or nonself-report) performance is quite consistent, but the moderating effect of achievement domain has yet to be tested. However, because some achievement goal (AG) measures are used exclusively for one particular achievement domain, we also explored AG measure as a potential moderator.

### Achievement Goal (AG) Measure as Moderator

The extant literature contains several established measures and operationalizations for achievement goals [12], [15], [17], [42], [45]–[50]; some of these are used exclusively for one particular achievement domain, which means that achievement domain and AG measure are partially confounded factors. Furthermore, the diversity in measures and operationalizations has created conceptual ambiguities, which might explain the mixed and inconsistent empirical results [51], [52]. For example, some measures define goals as standards for competence [53], [54], whereas other measures include items that refer to non-goal-relevant components, such as interest and affect [47], [49], [50]. Others [15] refer to broader, more general reasons for the pursuit of a certain goal (e.g., to prove to my teacher that I am the best; to impress my friends, etc.).

In their meta-analysis, Hulleman et al. [7] did address the moderating potential of AG measure. However, they compared only three AG measures: AGQ [12], [48] versus PALS [49] versus “other published AG measures”. They excluded studies “… in which goals were measured with statements of positive affect rather than goal-relevant language” (p. 430), including the (sport) studies in which the popular measure developed by Duda and Nicholls [47] was used. However, Hulleman et al. [7] included other measures of achievement goals that had “individual affective statements” (p. 430), such the PALS developed by Midgley et al. [49]. In addition, the VandeWalle [15] AG measure, which is widely used in the work domain, was collapsed into a broad, “other” category.

In contrast, in the present meta-analysis, we tested the relationships between goals and performance attainment separately for *all* established measures of achievement goals that have been used in the different achievement domains (for all measures, see Appendix S1). This approach enabled a full investigation of various aspects of the operationalization of achievement goals (e.g., standards, non-goal relevant components, reasons, or a combination of these), including the effect of type of items that were used on the AG measures. The demonstration of a moderating effect of AG measure would suggest that the mixed and inconsistent empirical results that have been obtained can be explained (at least partly) by the diversity in measures for achievement goals. An absence of statistical moderation due to AG measure would demonstrate consistency across conceptually different measures.

### Study Objectives

The purpose of the present study was to examine the relationships between self-reported achievement goals and nonself-report performance through meta-analysis, including the moderating potential of achievement domain. Accordingly, we first examined the overall correlations between each of the four types of achievement goal (MAp, PAp, PAv, and MAv) and performance. Next, we systematically tested whether relationships between achievement goals and performance attainment were moderated by achievement domain. In addition, we explored the moderating role of AG measure, a variable that is partially confounded with achievement goal domain, and we explored the moderating role of age, sex, nationality, and publication status. Where the number of available studies was sufficient, two-way interactions between moderators were examined.

## Method

### Sample of Studies

Both published and unpublished studies were identified using a variety of established meta-analytic search methods. First, a computerized web-based search of PsycINFO, Web of Science, and Dissertation Abstracts International up to January 1^st^, 2014 was conducted. These databases appeared to capture all articles of interest from the other more specialized databases, including Business Source Premier (work domain), ERIC (education domain), and Physical Education Index (sport domain). We searched the data bases by using the following key words (see also Appendix S2): *achievement goal, goal orientation, mastery goal, mastery approach goal, mastery-approach goal, approach goal, performance goal, performance approach goal, performance-approach goal, avoidance goal, performance avoidance goal, performance-avoidance goal, mastery avoidance goal, mastery-avoidance goal, learning goal, learning goal orientation, task goal, task goal orientation, prove goal, prove goal orientation, performance prove goal, performance prove goal orientation, ego goal, ego goal orientation, ability goal, performance,* and *performance attainment.* Second, we examined the reference lists of recent meta-analyses [5], [7], [9] and relevant review articles [8], [22], [43], [55]. Third, we browsed online databases (PsycINFO and Web of Science) using author names that are associated with specific measures for achievement goals (e.g., Duda, Elliot, Midgley, VandeWalle, etc.). Fourth, we contacted individual experts in the field and requested unpublished papers that could not be retrieved otherwise.

### Eligibility Criteria

To be included in the meta-analysis, studies had to meet the following criteria:

1. Achievement goals were measured at the individual level by using established measures. For the specific measures we included, see the section “Achievement Goal (AG) Measure” below (and Appendix S1). Adapted or customized versions were coded as “other” (see Appendix S3, Column L). Studies that measured goals at the group level, theoretical papers, and studies that induced achievement goals situationally were excluded.
2. The achievement goals could be categorized as MAp, PAp, PAv, or MAv. As discussed in the introduction, mastery-approach (MAp) goals focus on task-referenced or self-referenced improvement and accomplishments, whereas performance-approach (PAp) goals focus on performing better than others. Mastery-avoidance (MAv) goals are directed toward avoiding incompetence on the basis of a task-referenced or a self-referenced standard, whereas performance-avoidance (PAv) goals focus on avoiding failure relative to others.
3. The study could be coded as being conducted in one of the three achievement domains (education, work, or sports).
4. To exclude the possibility of same-source bias, only studies relying on nonself-report performance measures were included in our meta-analysis. Three studies that relied on self-report measures of performance were excluded [56]–[58]. For the nonself-report performance measure used in each individual study, see Appendix S3, Column E.
5. Zero-order correlations for the variables under scrutiny were reported, including statistically relevant information (e.g., sample size) that was sufficient to allow the computation of effect size statistics.

### Final Sample of Studies

In total, the final data set comprised 98 papers (for the references, see Appendix S3, Column A). Those papers contained 112 relevant samples (Column D), with a total of 33,983 participants, and 295 individual effect sizes (Column H). In this regard, it is important to note that in Hulleman’s et al. [7] meta-analysis, in which 243 studies were included (e.g., for examining relationships between different achievement goal measures), *only a portion* of these studies examined the relation between achievement goals and nonself-report performance, the focus of the current meta-analysis. How many studies Hulleman et al. [7] used exactly for this purpose is not clear. In Table 10 (p. 435), they report the inclusion of 98 studies for the PAp-performance outcomes correlation, 63 studies for the PAv-performance outcome correlation, 95 studies for the MAp-performance outcomes correlations, and 12 studies for the MAv-performance outcome correlations. However, most likely, these numbers refer to *effect sizes* rather than studies; studies rarely present just one goal-outcome correlation. Obviously, a couple of years later, in 2014, we could include more effect sizes than Hulleman et al. [7]: 106 effect sizes for PAp goals, 65 effect sizes for PAv goals, 103 effect sizes for MAp goals, and 31 effect sizes for MAv goals (see Appendix S3, Column H).

### Moderators

#### Domain

Each study was coded for the specific achievement domain (education, work, or sports) in which the achievement goals were assessed.

#### Achievement Goal (AG) Measure (for all measures, see Appendix S1)

In the *education domain*, the established measures for achievement goals are the trichotomous Achievement Goal Questionnaire (AGQ-3) and the 2×2 Achievement Goal Questionnaire (AGQ-4), which were developed by Elliot et al. [12],[42],[48], the Patterns of Adaptive Learning Survey (PALS) developed by Midgley et al. [49], and VandeWalle’s [15] goal orientation instrument.

The measures used in the *work domain* are the measures developed by VandeWalle [15] and Button’s et al. [45] goal orientation measure. We separately analyzed individual studies that used the Button et al. AG measure because it confounds approach and avoidance goals.

In the *sports domain*, the established measures include the dichotomous AG measures (performance versus mastery, or ego versus task) developed by Duda and Nicholls [47], [59] and Roberts et al. [50]. Based on the AGQ-4 measure, Conroy et al. [46] developed the 2×2 Achievement Goals Questionnaire for Sport (AGQ-S).

#### Additional moderators

To interpret possible moderating effects of AG measure, we first examined systematically the content of AG measures listed above. Two independent raters coded all individual goal items of each established measure for achievement goals. Items were coded as goal relevant if they contained language that referred to a standard (task, self, or others), a reason, or a mixture of standard and reason. For example, a “goal as standard” item was: “My aim is to perform well relative to other students”. A “goal as reason” item was: “An important reason I do my schoolwork is so that I do not embarrass myself”. An item that included a mixture of standard and reason was: “I want to do well in this class to show my ability to my family, friends, advisors, or others”. Items were coded as containing non-goal relevant language if they mentioned interest or enjoyment (e.g., “An important reason I do my schoolwork is because I enjoy it”), positive affect (e.g., “I feel most successful when a skill I learned feels right”), negative affect (e.g., “I am often concerned that I might not learn all there is to learn in this class”), or were worded as broad generic statements (e.g., “When I have difficulty solving a problem, I enjoy trying different approaches to see which one will work”). When items reflected combinations of standards, reasons, and non-goal-specific language, they were coded as mixed (e.g., “I would feel successful in school if I did better than most other students”). Agreement between the two coders was high: 86.3% (Cohen’s *k* = .81), with disagreements resolved through discussion to reach a consensus. For more detailed information about the coding of the items and the achievement goal measures, see Appendix S1.

As specified in Table 1, the 2×2 achievement goal measures (i.e., AGQ-4 and AGQ-S) have the largest percentage of achievement goal items that refer explicitly to standards of competence. In contrast, the PALS developed by Midgley et al. [49] and VandeWalle’s [15] goal orientation instrument contain a mixture of goal and non-goal relevant items (i.e., standard, reason, mixture, non-goal), and virtually all items in the measures of both Duda and Nicholls [47] and Roberts et al. [50] contain non-goal relevant language (i.e., mixture, non-goal).

**Table 1:** Goal Item Frequencies and Mean Total for Each AG measure.

	MAp				PAp				PAv				MAv		Mean AG measures			
AG measure	S	R	M	NG	S	R	M	NG	S	R	M	NG	S	M	S	R	M	NG
AGQ-3	50%		34%*	16%	85%		15%*		34%		16%^++^	34%			56.3%		27%	16.6%
											16%**							
AGQ-4	100%				100%				67%		33%^++^			100%**	66.7%		33.3%	
PALS		16%	34%*	50%		34%	33%^++^	33%		100%			33%	67%**	8.2%	37.5%	33.5%	20.7%
Duda & Nicholls			70%**	30%			100%**										85%	15%
Roberts et al.			34%**	66%			16%^++^										67%	33%
							84%**											
AGQ-S	100%				100%				100%					100%**	75%		25%	
VandeWalle	40%		40%**	20%	25%	25%	25%^++^				25%*	25%			21.6%	8.3%	55%	15%
							25%^+^				50%^+^							
	Mastery subscale				Performance subscale													
Button et al.	12%	12%	38%*	38%			38%^++^	62%							6%	6%	38%	50%

*Notes:*

S = Standard; R = Reason; M = Mixture; NG = Non-Goal. The Button et al. [45] measure is presented separately.

For the category “Mixture”, several combinations were possible: *Standard + Reason; **Standard + Non-goal; ^+^Reason+Non-goal; ^++^Standard + Reason + Non-goal.

In addition, in the meta-analysis, *age* was included as a continuous variable, and *sex* was calculated in terms of the proportion of women, represented by a score between 0 and 1. A small number of studies did not report relevant information on sex or age. When this was the case, the cells for those respective values were coded as missing (for sex) or approximated (for age). For example, the paper by Durik, Lovejoy, and Johnson [104] describes the sample as “college students from a large metropolitan area” in the United States of America. In this case, the age of the participants was approximated to match the average age of college students reported in similar studies.

*Nationality* was coded into four categories: 1 = *US/Canada*, 2 = *Europe*, 3 = *Asian*, and 4 = *other* (e.g., a sample of mixed ex-patriates). Finally, *publication status* was coded as two categories: 1 = *published*, and 2 = *unpublished* (e.g., dissertations, conference presentations, poster presentations).

### Nonself-report Performance Measure

Performance measures include grade point averages, mid-term exam scores, performance on subject-specific exams, such as Mathematics or Chemistry, and class performance as assessed by teachers (*education domain*), sales performance and supervisor-rated job performance (*work domain*), and performance on particular exercises, ranking in tournaments, outcomes of competitions, and assessments by coaches or trainers (*sports domain*); see Appendix S3 (Column E) for the nonself-report performance measure used in each individual study.

### Statistical Methods

All data were analyzed in SPSS using macros for meta-analysis [60]. For each study in the meta-analysis, an effect size (*r*) was obtained between a specific achievement goal and performance attainment.

Positive and negative relationships between achievement goals and performance are reflected by positive and negative effect sizes, respectively. Almost all papers that were included in the meta-analysis reported at least two correlations per study (e.g., MAp goals and performance, and PAp goals and performance; see Appendix S3, column G). To meet the statistical assumption of effect size independence [4], analyses were conducted on four separate data sets of studies that reported a correlation between: (1) MAp goals and performance; (2) PAp goals and performance; (3) PAv goals and performance; and (4) MAv goals and performance. Following the recommendations of Lipsey and Wilson [4] and Wilson [60], effect sizes were Fisher-Z transformed, and inverse variance weights were used during the data analysis. All statistical analyses were performed under a random-effects model, which assumes that all effect sizes are sampled randomly from a population of possible effect sizes, with sampling error being the variance of both random effects and estimated variance [61]. With this approach, inference tests tend to be more conservative.

The overall rate of agreement between the two independent coders with respect to effect size statistics and moderators (domain, AG measure, age, sex, nationality, and publication status) was not very high, but acceptable: 79.5% (Cohen’s *k* = .58). Important to note is that the data were analyzed after disagreements between coders had been resolved through discussion to reach a consensus.

### Tests for Publication Bias

Following the recommendations of Ferguson and Brannick [62], we used a tandem procedure for detecting publication bias: (1) We calculated Rosenberg’s [63] fail-safe *N* to determine the number of “file-drawer” studies necessary to reduce the combined significance to alpha = .05. (2) We examined funnel plots of effect sizes X standard error and used Egger’s regression method to quantify possible biases in the funnel plot. (3) To estimate how much observed results could be influenced by bias, we implemented Duval and Tweedie’s [64] trim-and-fill procedure. Analyses were conducted with the metafor package in R, version 1.9–2 [65].

Again, the four achievement goals were considered separately. For PAp goals, Egger’s regression suggested a slight possible asymmetry (*p = *.02), but the fail safe *N = *11,548 with *k = *106 and trim and fill statistics flagged no publication bias concerns. Visual inspection of plots suggested no obvious causes for concern about asymmetry, but the variability of effects was rather high (which negatively affects bias detection).

For MAp goals, neither the fail safe *N = *16824 with *K = *103, nor Egger’s regression (*p = *.62) indicated bias. But the trim and fill procedure did identify some asymmetry: three effect sizes were estimated on the left side due to an apparent underreporting of effects around zero. Compensating for this would make only little difference to reported effects (r2 difference = .0074).

For PAv goals none of the indicators showed any sign of possible bias (fail safe *N = *6141 with *k = *65, Egger’s regression *p = *.93, trim and fill *k = *0).

Finally, there was only a small number of *k = *21 studies reporting MAv goals. Because of the small number, effects should be interpreted with caution. Indicators suggested bias was not an issue (fail safe *N = *83, Egger’s regression *p = *.87, trim and fill *K = *0).

### Influential Data Points

Next, we checked for influential data points for each of the four achievement goals separately. We used the influence function in metafor to identify potential outliers based on several indicators (Cook’s distance, hatvalues, DFITTS, DFbeta’s, and covariance ratio’s). We examined potential outlier’s study and sample characteristics. We also removed potential outliers to determine impact on reported results. We concluded that none of the influential data points was either suspect or influential enough to justify exclusion; results remained essentially unchanged when influential data points were removed. There was one exception to this that should be noted. From the 21 studies examining mastery-avoidance goals, the study by Dysvik and Kuvaas [66] in the work domain had an unusual positive effect size (*r = *.16). We decided to retain this one influential data point for the analyses reported below, partly because this is the only study in the work domain (suggesting there may be systematic reasons for it being different from the other findings), because the overall *k* is low, and because including the study did not affect the conclusions drawn (e.g., exclusion of this effect would have resulted in an overall difference in aggregated r of only.018).

## Results

### Descriptive Statistics

The final data set comprised 98 papers (for the references, see Appendix S3, Column A), of which 78 (79.6%) were from the educational domain, 13 (13.3%) from the sports domain, and 7 (7.1%) from the work domain.

The AGQ-3 [48] was used in 17 samples (15.2%), the AGQ-4 [12] in 13 samples (11.6%), the PALS measure [49] in 16 samples (14.3%), Duda and Nicholls’ [47] measure in 5 samples (4.5%), the Roberts et al. [50] measure in 3 samples (2.7%), the AGQ-S [46] in 8 samples (7.1%), the VandeWalle’s [15] measure in 13 samples (11.6%), and the Button et al. [45] measure in 12 samples (10.7%). The remaining 25 samples (22.3%) in which adapted and customized existing measures were used, were coded as “other” (see Appendix S3, Column L).

The percentage of women was 53.1%, and the participants were mostly of US or Canadian nationality (59.0%), followed by European (23.0%), Asian (10.8%), and other nationalities (7.2%).

### General Effects

Following the recommendations of Wilson [60], we first conducted tests for relevant basic central tendency statistics, such as mean effect size, *Z*-tests, and homogeneity testing. As shown in Table 2, overall positive correlations were found between MAp goals and performance (*r*_MAp_ = .14, *Z* = 11.78, *p*<.001), and between PAp goals and performance (*r*_PAp_ = .10, *Z* = 7.93, *p*<.001). Overall negative correlations were observed between PAv goals and performance (*r*_PAv_ = −.13, *Z* = −10.39, *p*<.001) and MAv goals and performance (*r*_MAv_ = −.07, *Z* = −2.23, *p* = .026). The significant values of within-class variance (*Q*_w_, see Table 2) for the effect sizes indicated heterogeneity among effect sizes in the data sets, which signaled the potential presence of moderators [4]. The results of the moderator analyses for each categorical moderator variable (domain, AG measure, nationality, and publication status) are presented in Table 3 (MAp goals), Table 4 (PAp goals), Table 5 (PAv goals), and Table 6 (MAv goals). The results for the moderator variables age and sex are presented in Table 7. Below, we first discuss moderation by achievement domain, and next, the effects of the additional moderators.

**Table 2:** Results for the Overall Achievement Goal-Performance Correlations.

	*r*_w_ with Performance	95% CI	*k*	*Z*	*Q*_w_	Effect size range
MAp goals	.14	.12, .16	103	11.78**	394.47**	−.11, .41
PAp goals	.10	.07, .12	106	7.93**	465.49**	−.38, .38
PAv goals	−.13	−.16, −.11	65	−10.39**	193.47**	−.31, .27
MAv goals	−.07	−.13, −.01	31	−2.23*	129.64**	−.29, .17

Notes:

*r*_w_ = effect size correlation coefficients, CI = confidence intervals, *k* = number of effect sizes,

*Z* = z-score, *Q*_w_ = within-class goodness-of-fit statistics.

*p<.05; **p<.01.

**Table 3:** Results of the Moderator Analyses: Mastery-Approach Goals.

	Between class effects						
Variable	*Q*_b_	*df*	*r*_w_	95% CI	*k*	*Z*	Homogeneity within class (*Q*_w_)
**Domain**	9.60**	2					
1. Education			.13^a^	.10, .15	72	9.57**	77.21
2. Work			.27^b^	.18, .35	6	5.80**	1.50
3. Sports			.17^ab^	.10, .23	13	5.10**	8.75
**AG measure**	6.58	8					
1. AGQ-3			.12	.06, .18	15	4.06**	9.76
2. AGQ-4			.12	.05, .19	10	3.35**	3.04
3. PALS			.17	.11, .23	15	5.88**	28.24*
4. Duda & Nicholls			.13	.02, .23	5	2.40*	1.84
5. Roberts et al.			.16	.02, .30	3	2.26*	2.98
6. AGQ-S			.19	.10, .27	7	4.05**	3.34
7. VandeWalle (work)			.28	.21, .34	4	7.69**	5.23
8. VandeWalle (education)			.13	.06, .20	9	3.75**	15.78
9. Other (only education)			.10	.06, .15	23	4.30**	20.35
**Nationality**	14.41**	3					
1. US/Canadian			.11^a^	.08, .14	59	7.47**	56.92
2. European			.19^b^	.14, .23	20	7.50**	18.49
3. Asian			.21^b^	.14, .28	9	5.98**	8.09
4. Other			.23^b^	.09, .35	3	3.21**	.61
**Publication status**	4.20*	1					
1. Published			.15^a^	.12, .17	82	11.45**	76.25
2. Not Published			.06^b^	−.01, .14	9	1.63	8.18

*Notes:*

Cells not sharing a common superscript differ significantly (*p*<.05) from each other.

*r*_w_ = correlation coefficient, CI = confidence intervals, *k* = number of effect sizes, *Z* = z-score.

*p<.05; **p<.01.

**Table 4:** Results of the Moderator Analyses: Performance-Approach Goals.

	Between class effects						
Variable	*Q*_b_	*df*	*r*_w_	95% CI	*k*	*Z*	Homogeneity within class (*Q*_w_)
**Domain**	1.60	2					
1. Education			.10	.08, .13	75	7.28**	73.65
2. Work			.13	.02, .22	6	2.44*	3.06
3. Sports			.15	.08, .22	14	4.24**	34.45**
**AG measure**	11.40	8					
1. AGQ-3			.09^a^	.01, .15	17	3.10**	12.14
2. AGQ-4			.19^b^	.12, .24	13	5.80**	8.26
3. PALS			.07^a^	.01, .13	16	2.40*	25.63*
4. Duda & Nicholls			.12^a^	.01, .23	5	2.22*	7.04
5. Roberts et al.			.07^a^	−0.09, .20	3	.79	7.26*
6. AGQ-S			.18^b^	.09, .26	8	3.85**	21.27**
7. VandeWalle (work)			.14^ab^	.06, .21	4	3.49**	5.88
8. VandeWalle (education)			.04^a^	−.03, .10	7	1.14	4.44
9. Other (only education)			.11^a^	.06, .16	22	4.31**	20.84
**Nationality**	1.16	3					
1. US/Canadian			.10	.07, .13	60	6.28**	51.43
2. European			.12	.07, .17	23	4.68**	52.67**
3. Asian			.13	.05, .21	9	3.30**	4.47
4. Other			.15	−.01, .29	3	1.87	2.17
**Publication status**	3.02	1					
1. Published			.12	.09, .14	85	8.93**	102.18
2. Not Published			.05	−.03, .13	10	1.28	11.33

*Notes:*

Cells not sharing a common superscript differ significantly (*p*<.05) from each other.

*r*_w_ = correlation coefficient, CI = confidence intervals, *k* = number of effect sizes, *Z* = z-score.

*p<.05; **p<.01.

**Table 5:** Results of the Moderator Analyses: Performance-Avoidance Goals.

	Between class effects						
Variable	*Q*_b_	*df*	*r*_w_	95% CI	*k*	*Z*	Homogeneity within class (*Q*_w_)
**Domain**	6.23*	2					
1. Education			−.14^a^	−.17, −.11	55	−10.10**	58.26
2. Work			−.20^a^	−.31, −.08	3	−3.33**	.53
3. Sports			−.04^b^	−.12, .05	7	−.86	9.96
**AG measure**	10.53	6					
1. AGQ-3			−.15	−.20, −.10	17	−5.88**	26.61*
2. AGQ-4			−.19	−.25, −.14	12	−6.50**	8.59
3. PALS			−.11	−.16, −.05	14	−3.83**	5.07
4. AGQ-S			−.04	−.13, .05	7	−.83	9.70
5. VandeWalle (work)			−.18	−.24, −.12	2	−5.51**	1.08
6. VandeWalle (education)			−.08	−.18, .01	6	−1.68	10.48
7. Other (only education)			−.13	−.20, −.06	7	−3.61**	4.03
**Nationality**	1.47	3					
1. US/Canadian			−.14	−17, −.11	37	−8.08**	39.83
2. European			−.14	−.19, −.09	17	−5.77**	16.83
3. Asian			−.10	−.17, −.03	8	−2.76**	9.43
4. Other			−.09	−.23, .04	3	−1.39	6.83*
**Publication status**	1.41	1					
1. Published			−.14	−.17, −.11	56	−10.17**	66.48
2. Not Published			−.10	−.16, −.03	9	−2.78**	8.43

*Notes:*

Cells not sharing a common superscript differ significantly (*p*<.05) from each other.

*r*_w_ = correlation coefficient, CI = confidence intervals, *k* = number of effect sizes, *Z* = z-score.

*p<.05; **p<.01.

**Table 6:** Results of the Moderator Analyses: Mastery-Avoidance Goals.

	Between class effects						
Variable	*Q*_b_	*df*	*r*_w_	95% CI	*k*	*Z*	Homogeneity within class (*Q*_w_)
**Domain**	23.46**	1					
1. Education			−.13^a^	−.17, −.08	13	−5.43**	17.35
2. Sports			.02^b^	−.06, .09	7	.41	2.66
**AG measure**	3.76	2					
1. AGQ-4			−.12	−.21, −.03	10	−2.67**	8.76
2. PALS			−.07	−.21, .07	4	−1.04	1.88
3. AGQ-S			.02	−.09, .14	7	.40	1.36
**Nationality**	12.26**	2					
1. US/Canadian			−.16^a^	−.24, −.08	6	−3.83**	3.24
2. European			.03^b^	−.05, .11	8	.77	7.97
3. Asian			−.10^a^	−.18, −.02	6	−2.35*	5.25
**Publication status**	5.05*	1					
1. Published			−.05^a^	−.11, .01	19	−1.61	13.55
2. Not Published			−.29^b^	−.46, −.09	2	−2.82**	0.01

*Notes:*

Cells not sharing a common superscript differ significantly (*p*<.05) from each other.

*fr*_w_ = correlation coefficient, CI = confidence intervals, *k* = number of effect sizes, *Z* = z-score.

*p<.05; **p<.01.

**Table 7:** Simple Regressions of the Moderators Age and Sex on Effect Sizes.

	*r*_MAp_			*r*_Pap_			*r*_PAv_			*r*_MAv_		
Simple regression	*B*	*Z*	*R* ^2^	*B*	*Z*	*R* ^2^	*B*	*Z*	*R* ^2^	*B*	*Z*	*R* ^2^
Age	.12	1.22	.01	.12	1.33	.01	−.08	−.74	.007	.23	.11	.05
Sex	.05	.50	.003	.04	.45	.002	−.13	−1.09	.02	−.47	−2.14	.22

*Note:*

*B = *Standardized regression coefficient, *Z* = z-score.

### Moderation by Achievement Domain

The overall positive correlation between MAp goals and performance attainment (see Table 2) was qualified by achievement domain, *Q*_b_(2) = 9.60, *p* = .008 (see Table 3). Follow-up significance testing revealed that the correlation between MAp goals and performance was significantly higher in the work domain (*r = .*27) relative to the education (*r = *.13) and sports domains (*r = *.17).

In contrast, Table 4 shows that the overall positive correlation between PAp goals and performance was not qualified by achievement domain. However, inspection of the confidence intervals suggests that particularly in the work domain, PAp goals (*r*_PAp_ = .13, see Table 4) seem to be less strongly related to performance than MAp goals (*r*_MAp_ = .27, see Table 3). Note that the four types of achievement goals were analyzed separately to meet the assumption of effect size independence. Hence, differences in effect sizes between different goals (e.g., PAp versus MAp) cannot be tested directly; we can only speculate on the differences in effect sizes and on their meaning.

Table 5 shows that achievement domain moderated the overall negative correlation between PAv goals and performance (*Q*_b_ = 6.23, *p = *.044). Specifically, the correlation between PAv goals and performance was negative in the work (*r = *−.20) and education domains (*r* = −.14), and not significant in the sports domain (*r* = −.04).

With regard to MAv goals, Table 6 shows that different correlations were found in the education and sports domains, *Q*_b_ (1) = 23.46, *p*<.001. The correlation between MAv goals and performance was negative in the education domain (*r*_MAv_ = −.13), but nonsignificant in the sports domain. In this analysis, the work domain was not included because only one study [66] reported a link between MAv goals and job performance.

Additional patterns of interest were observed in the sports domain, in which approach goals were correlated significantly and positively with performance attainment, whereas avoidance goals were found to be unrelated to performance. In contrast, in the education domain, the goal–performance relationships were approximately equally as strong and significant for each of the four types of goal: positive for approach goals, negative for avoidance goals. In the work domain, approach goals (MAp goals in particular) were related positively to performance, whereas PAv goals were related negatively to performance (in the work domain, data on MAv goals were not available).

### Additional Moderators

#### Achievement goal (AG) measure

The overall correlations between either achievement goal and performance (see Tables 3–6) were not qualified by AG measure. However, as shown in Table 4, follow-up contrasts analyses revealed significantly higher correlations between PAp goals and performance in studies that used the AGQ-4 (primarily in the classroom) and the AGQ-S (on the sports field). These PAp subscales only comprise goal-relevant items that refer explicitly to other-referenced standards of competence (see Table 1).

As indicated earlier, the performance orientation subscale developed by Button et al. [45] cannot be categorized within the 2×2 framework because in this measure, PAp and PAv goals are confounded. For the sake of completeness, we conducted a separate meta-analysis among the 12 samples in which this measure was linked to performance attainment [45], [67]–[73]. In line with the general pattern (see Table 2), this analysis revealed a significant, positive correlation between MAp goals (referred to as “learning goals” by the authors) and performance attainment (*r*_MAp_ = .13, *p*<.001). Not surprisingly, however, the observed correlation between undifferentiated performance goals and performance attainment was *r* = −.02, *p* = .33. The correlation coefficients of opposite valence that were observed in general for PAp and PAv goals in other studies (*r*_PAp_ = .10 and *r*_PAv_ = −.13, see Table 2) apparently average to zero when a measure of undifferentiated performance goals is used. This was exactly why the valence dimension was added to the conceptualization of achievement goals [14]–[16], [74].

#### Nationality

Correlations between both MAp goals and performance, *Q*_b_ (3) = 14.41, *p = *.002 (see Table 3) and MAv goals and performance, *Q*_b_ (2) = 12.26, *p = *.007 (see Table 6) were moderated by nationality. With regard to MAp goals (see Table 3), the strongest positive correlations were observed in Asian samples (*r* = .21) and “other” samples (*r* = .23). A weaker correlation was found in US/Canadian samples (*r* = .11). For MAv goals (see Table 6), no significant link was found between MAv goals and performance in European samples, whereas MAv goals and performance were correlated negatively in Asian samples (*r* = −.10) and US/Canadian samples (*r*_MAv_ = −.16).

*Publication status* significantly moderated the correlations between MAp goals and performance (*Q*_b_ = 4.20, *p = *.040) and between MAv goals and performance (*Q*_b_ = 5.05, *p = *.025). In contrast to the overall pattern, in unpublished studies, MAp goals were not significantly related to performance (*r = *.06). The overall negative correlation between MAv goals and performance was only observed in unpublished studies. However, because of the low number of studies, this latter result should be interpreted with caution.

*Age* and *sex* were regressed on the correlations between achievement goals and performance in four separate analyses. As shown in Table 7, neither age nor sex emerged as a significant moderator in any of the four regression models (*p*s>.10).

### Multivariate Analyses

The number of studies in each cell allowed the testing of two-way interactions between domain, age, and sex only on the relationship between approach goals (MAp and PAp) and performance attainment. Following the recommendations of Aiken and West [75], the independent categorical variable “domain” was dummy coded. The educational domain, which had the largest number of studies, was taken as the reference group. No interaction effects emerged between age and sex, age and domain, or sex and domain (*p*s>.10).

## Discussion

In the present meta-analysis, we systematically reviewed 98 papers that had been published up to January 1^st^, 2014, which comprised a total of 295 individual effect sizes and 33,983 participants. Overall, the relationships between self-reported approach goals (either MAp or PAp) and nonself-report performance were positive, and the relationships between avoidance goals (either PAv or MAv) and performance were negative. These findings are in line with previous meta-analyses [5], [7], [9].

### Achievement Domain as a Moderator

Our findings extend the scope of previous work by showing that relationships between achievement goals and performance attainment can differ across the domains of education, work, and sports. Specifically, the robust, positive link between MAp goals and performance appears to be particularly strong in the work domain. An explanation for this finding may be that more than performance at school (i.e., exam performance) or on the sports field (i.e., scores), job performance includes extra-role behavior, that is, non-prescribed organizationally beneficial behaviors and gestures [23], [76]. Specifically, job performance is a broad and complex construct comprising two fundamentally different aspects: in-role job performance mandated by an organization, and extra-role performance. Because MAp goals (and intrinsic work motivation) are important motivational sources for extra-role behavior in particular [23], the MAp goal – performance relationship may be particularly strong among workers relative to students and athletes.

In the education and work domains, PAv goals were negatively related to performance. Indeed, the extant achievement goal literature suggest that PAv goals are consistently associated with negative outcomes such as anxiety, disorganized habits, negative affect, help-avoidance, disinterest, and low performance [2], [54], [55]. However, across studies in the sports domain, we did not observe a negative link between PAv goals and performance (see Table 5). Given that competitiveness and social comparison are inherent to most games and sports [77]–[80], a performance goal-oriented sports climate may better fit with individuals’ PAv goals. That is, individuals with PAv goals may not necessarily “feel bad” in a sports context, which may mitigate a decrease in task focus, effort, and persistence, and ultimately, performance deterioration [81], [82]. Furthermore, in a sports context, a performance-avoidance goal may not have such a negative connotation because not performing worse than others, or not losing (i.e., a draw), may be perceived as a great achievement or a desired outcome, for example, because the opponent is considered as much stronger, or because not losing may be sufficient to qualify for the next round in a tournament or to become league champion.

Similarly, in the sports domain, MAv goals were unrelated to performance, whereas in the education domain, the MAv–performance correlation was negative (see Table 6). In educational settings, in which learning, development, and improvement are typically emphasized [22], the goal of avoiding not learning, developing, and improving might evoke low perceptions of competence, negative affect, and cognitive anxiety. For example, Sideridis [83] found that MAv goals in particular interfered with students’ emotional self-regulation during class presentations and exams. In contrast, in a sports context, within which competitive outcomes are more salient [77]–[80], it might be more likely that athletes perceive a performance at their typical level (i.e., not performing worse than before) to be sufficient for a win or a particular rank. However, given the novelty of the MAv goal construct and the relatively low number of studies on MAv goals that has been conducted to date, these findings should be interpreted with caution. Similarly noteworthy is the observation that when the differences between the achievement domains were considered, almost all the effects within each domain were quite homogeneous (as evidenced by a nonsignificant homogeneity within class).

### Additional Moderators

By including *all* established achievement goal measures (see Appendix S3, Column L), we found hardly any evidence that AG measure moderated the link between achievement goals and performance. For example, the negative relationship between PAv goals and performance held for AG measures that comprised items framed as standards (e.g., “My aim is to avoid doing worse than others”), as reasons (e.g., “One of my main goals is to avoid looking like I can’t do my work”), or as negative affect (e.g., “My fear of performing poorly in this class is what often motivates me”). Similarly, the negative relationship between MAv goals and performance held for AG measures that consisted of items framed as standards (e.g., “My aim is to avoid learning less than I possibly could”) or as negative affect (e.g., “Sometimes I’m afraid that I may not perform as well as I’d like”). This absence of statistical moderation due to AG measure suggests that the relationships between self-reported avoidance goals (either PAv or MAv) and nonself-report performance is negative, regardless of the type of AG measure used.

We only found some evidence that the positive correlations between PAp goals and performance were particularly strong in studies that used the AGQ-4 (primarily in the classroom) and the AGQ-S (on the sports field). These PAp subscales exclusively comprise goal-relevant items that refer explicitly to other-referenced standards of competence (see Table 1). Conversely, when the percentage of goal-relevant items decreased, correlations between PAp goals and performance decreased as well [7]. For example, correlations between PAp goals and performance were lower in studies that used the AGQ-3 [48], the Duda and Nicholls [47] AG measure, the Roberts et al. [50] AG measure, and the PALS [49], which all contain goal-related expressions of emotion or feeling (e.g., “I would feel successful…”), or items that are relevant to appearance (e.g., “…to show my ability…”, “…to show that I am smarter…”). In particular, emotional forecasting and concerns about self-presentation, that is, processes that tie the individual’s self-worth closely to performance attainment, might shift the attention away from the task itself, and accordingly, harm performance attainment [7], [84]. Thus, although the relations between PAp goals and performance are positive across the different modes of operationalization, we found some evidence that they are especially strong when items refer explicitly to other-referenced standards of competence. However, because statistical moderation due to AG measure was largely absent, we may conclude that the observed moderation of achievement domain cannot be explained by AG measure, the moderator that is partially confounded with achievement domain.

Furthermore, age and sex did not emerge as significant moderators. In contrast, nationality and publication status moderated the relationship between mastery-based goals (either MAp or MAv; see Table 3 and 6, respectively) and performance attainment. Most notably, mastery-based goals seem to be less beneficial among US/Canadian people. That is, in samples from these countries, MAp goals are less positively related to performance whereas MAv goals are more negatively related to performance. More cross-cultural research is obviously needed to clarify these unexpected findings. Similarly, publication status moderated the links between mastery-based goals and performance attainment. Only in published studies, MAp goals were significantly related to performance whereas significant negative correlations between MAv goals and performance were reported particularly in unpublished studies. However, because of the low number of studies, the results on MAv goals should be interpreted with caution.

### Limitations and Future Directions

The current work is not without limitations. First, the smaller number of studies in some achievement domains prevented the testing of higher-order interactions between the factors. Future meta-analyses will address this issue by adding the studies that will appear in years to come. Second, achievement domain and AG measure were partially confounded. Although the absence of a moderation effect of AG measure suggests that the observed differences between achievement domains can indeed be ascribed to domain rather than AG measure, an important direction for future research may be to use the same AG measure across the different achievement domains. By doing so, the moderating effect of achievement domain can be ascribed more unequivocally to differences between the achievement domains. To optimize conceptual clarity, such a measure could be stripped of any non-goal-relevant language and be rooted exclusively in the two fundamental components of competence: how competence is defined and how it is valenced [12], [17]. As discussed elaborately by Elliot et al. [85], three basic evaluative standards can be identified in the determination of whether one is doing well or poorly: task, self, and other. These evaluative standards can be pursued as a positive, desirable possibility (i.e., success), that is, individuals may be aiming at doing a task correctly, doing better than before, or doing better than others, respectively. Alternatively, the standard may be considered as being a negative, undesirable possibility (i.e., failure) that should be avoided. Specifically, an individual’s goals may be to avoid doing a task incorrectly, to avoid doing worse than before, or to avoid doing worse than others, respectively. Elliot et al. [85] argued and demonstrated that the distinction between these three different standards for competence, and how they are valenced, is warranted both theoretically and empirically. For example, when discussing their idea to divide mastery goals into task-based and self-based goals, Elliot et al. [85] pointed out that a task-referenced standard necessitates only the ability to represent the task, whereas a self-referenced standard requires the ability to evaluate outcomes progressively (some of which are not immediately present), and to use abstract information to separate self-based striving from ongoing engagement in the task. They successfully developed and validated a 3×2 achievement goal questionnaire for an educational context (see Elliot et al. [85], p. 648). By adapting the wording of the items, this measure can be applied to the work or sports context, either at a specific level (i.e., work assignment/project or competition/exercise) or a broader, more general level (i.e., one’s studies, work, or sports; see Appendix S4 and S5, respectively). Furthermore, to facilitate the comparison of results across achievement domains and methodologies, the same conceptualization of competence (i.e., definition versus valence) should be used in experimental studies and intervention studies. In the long term, this will create a collective database for future meta-analyses that can address the limitations of the present research, and accordingly, further broaden and deepen our understanding of the relationship between achievement goals and performance attainment.

### Concluding Remarks

A robust and consistent finding across achievement domains and conceptualizations of achievement goals is that approach goals (either MAp or PAp) are positively related to performance, whereas avoidance goals (either PAv or MAv) are negatively related to performance. Nevertheless, with the aim of performance enhancement, achievement goal-based interventions should focus in particular on promoting MAp goals (rather than PAp goals) for several reasons. First, in many achievement settings and contexts (often explicitly on the sports field, but also in the classroom and the workplace), visible and public performance evaluations are typically based on comparisons with others [86]–[88]. Hence, even among mastery goal individuals there is a consistent, dominant reliance on social comparisons over temporal comparisons in their performance self-evaluations [89]. Promoting PAp goals would strengthen individuals’ reliance on social comparison even more. Second, in general, the pursuit of MAp goals is considered to be the ideal type of competence-based regulation [2], [14]. For example, individuals who hold MAp goals have been found to have high levels of need for achievement [48], intrinsic motivation [90], task interest [43], and agreeableness and conscientiousness [70], [91]. Third, MAp goals tend to promote prosocial behavior, such as tolerance for opposing views [92] and sharing resources with others [93], [94]. In contrast, PAp goals show a mixed-valence profile, probably because these hybrid goals contain both a positive component (approach orientation) and a negative component (performance orientation) [12]. For example, on the positive side, individuals who hold PAp goals tend to have high levels of achievement motivation [48], conscientiousness [95], and positive affectivity [54]. However, PAp goals can involve some costs in terms of interest [43], anxiety, worry, negative affect [12], [14], dissatisfaction [96], and neuroticism [91], [97]. Furthermore, PAp goals tend to elicit unethical behaviors such as thwarting behavior and less accurate information giving [98] and cheating [99]. Thus, although PAp goals have consistent positive effects on performance attainment, undesirable social and ethical consequences of these goals might caution practitioners against their promotion.

A MAp goal-oriented motivational climate that directs individuals towards task-referenced or intrapersonal standards can be created, for example, by emphasizing evaluation more in terms of progress and effort, by defining success more in terms of improvement, by accepting errors or mistakes as part of the learning process, particularly in training programs, and by emphasizing enjoyment, interest, and collaboration [100], [101]. Important to note is that an emphasis on MAp goals does not imply the absence of interpersonal standards, social comparison, or competition. In contrast, in any achievement setting, interpersonal evaluation is apparent [89] and even necessary [102]. The key is the extent to which managers, teachers, and coaches emphasize other-referenced versus task-referenced or self-referenced standards [89], and whether they link task-referenced or self-referenced performance evaluations to (non)material rewards. This insight might help to educate effective, successful, and ethical workers, students, and athletes alike.

## Supporting Information

Appendix S1 (DOCX)

Appendix S2 (DOCX)

Appendix S3 (XLS)

Appendix S4 (DOCX)

Appendix S5 (DOCX)

Checklist S1 (DOCX)

Protocol S1 **PRISMA 2009 Flow Diagram Meta-Analysis.**
(DOC)
