# Supplementary material for: A Meta-Analysis of Self-Reported Achievement Goals and Nonself-Report Performance across Three Achievement Domains (Work, Sports, and Education)
Source: PLoS One. 2014 Apr 3;9(4):e93594. doi: 10.1371/journal.pone.0093594 (PMC3974764; doi:10.1371/journal.pone.0093594)
Supplement: Appendix S1 — (DOCX) [file pone.0093594.s001.docx]

**Appendix S1**

Details about the coding of the items from the established achievement goal measures included in the meta-analysis.

| Category | Description |
| --- | --- |
| 1. Goal as Standard | - 1. *Task-referenced* standard, focusing on an event, either approach (e.g., getting an answer correct, doing the task as well as possible), or avoidance (e.g., not getting an answer wrong). |
|  | - 1. *Self-referenced* standard, focusing on learning, and improving compared to one’s past performance, or future potential performance, either approach (e.g., to perform better than I did before, to learn), or avoidance (e.g., to avoid performing worse than I did before, or avoid not learning). |
|  | - 1. *Other-referenced* standard, focusing on performance relative to significant others, or on performance relative to a normative distribution of others (e.g., class grade), either approach (e.g., to do better than others), or avoidance (e.g., not to do worse than others). |
| 1. Goal as Reason | - 1. The broader, more general purpose for why one pursues a certain standard, either approach (e.g., to show my ability, to get a reward) or avoidance (to avoid the shame of failure). Reasons (behind a standard) can be exhaustive in scope and breadth. |
| 1. Non-Goal | - 1. Goal-related affect, or interest (e.g., I feel successful when …, I am interested in …, I enjoy …) |
|  | - 1. Non-goal language: comparisons (e.g., I prefer to do things that I do well rather than things that I do poorly), appearance-relevant language (e.g., The opinions others have about how well I can do certain things are important to me), decision making and choices (e.g., I prefer course material that arouses my curiosity, even if it is difficult to learn). |

*Note*:

Individual items may pertain to more than one category, e.g. a combination of standard and reason (e.g., I want to do better than others, *because* I want to impress my parents).

**The trichotomous Achievement Goal Questionnaire developed by Elliot and Church [48]**

Mastery Goal Subscale

|  | Goal | | Non-goal |
| --- | --- | --- | --- |
| Item | Standard | Reason |  |
| I want to learn as much as possible from this class | · |  |  |
| It is important for me to understand the content of this course as thoroughly as possible | · |  |  |
| I hope to have gained a broader and deeper knowledge of [subject] when I am done with this class | · | · |  |
| I desire to completely master the material presented in this class | · |  |  |
| In a class like this, I prefer course material that arouses my curiosity, even if it is difficult to learn |  |  | · |
| In a class like this, I prefer course material that really challenges me so I can learn new things | · | · |  |

Performance-Approach Goal Subscale

|  | Goal | | Non-goal |
| --- | --- | --- | --- |
| Item | Standard | Reason |  |
| It is important to me to do better than the other students | · |  |  |
| My goal in this class is to get a better grade than most of the students | · |  |  |
| I am striving to demonstrate my ability relative to others in this class | · |  |  |
| I am motivated by the thought of outperforming my peers in this class | · |  |  |
| It is important to me to do well compared to others in this class | · |  |  |
| I want to do well in this class to show my ability to my family, friends, advisors, or others | · | · |  |

Performance-Avoidance Goal Subscale

|  | Goal | | Non-goal |
| --- | --- | --- | --- |
| Item | Standard | Reason |  |
| I often think to myself, "What if I do badly in this class?'' | · |  |  |
| I worry about the possibility of getting a bad grade in this class | · |  | · |
| My fear of performing poorly in this class is often what motivates me | · | · | · |
| I just want to avoid doing poorly in this class | · |  |  |
| I'm afraid that if I ask my TA or instructor a "dumb" question, they might not think I'm very smart |  |  | · |
| I wish this class was not graded |  |  | · |

**The 2 x 2 Achievement Goal Questionnaire developed by Elliot and McGregor [12]**

Mastery-Approach Goal Subscale

|  | Goal | | Non-goal |
| --- | --- | --- | --- |
| Item | Standard | Reason |  |
| I want to learn as much as possible from this class | · |  |  |
| It is important for me to understand the content of this course as thoroughly as possible | · |  |  |
| I desire to completely master the material presented in this class | · |  |  |

Performance-Approach Goal Subscale

|  | Goal | | Non-goal |
| --- | --- | --- | --- |
| Item | Standard | Reason |  |
| It is important for me to do better than other students | · |  |  |
| It is important for me to do well compared to other students in this class | · |  |  |
| My goal in this class is to get a better grade than most of the other students | · |  |  |

Performance-Avoidance Goal Subscale

|  | Goal | | Non-goal |
| --- | --- | --- | --- |
| Item | Standard | Reason |  |
| I just want to avoid doing poorly in this class | · |  |  |
| My goal in this class is to avoid performing poorly | · |  |  |
| My fear of performing poorly in this class is often what motivates me | · | · | · |

Mastery-Avoidance Goal Subscale

|  | Goal | | Non-goal |
| --- | --- | --- | --- |
| Item | Standard | Reason |  |
| I worry that I may not learn all that I possibly could in this class | · |  | · |
| Sometimes I’m afraid that I may not understand the content of this class as thoroughly as I’d like | · |  | · |
| I am often concerned that I may not learn all that there is to learn in this class | · |  | · |

**The Revised 2 x 2 Achievement Goal Questionnaire developed by Elliot and Murayama [42]**

Mastery-Approach Goal Subscale

|  | Goal | | Non-goal |
| --- | --- | --- | --- |
| Item | Standard | Reason |  |
| My aim is to completely master the material presented in this class | · |  |  |
| I am striving to understand the content of this course as thoroughly as possible | · |  |  |
| My goal is to learn as much as possible | · |  |  |

Performance-Approach Goal Subscale

|  | Goal | | Non-goal |
| --- | --- | --- | --- |
| Item | Standard | Reason |  |
| My aim is to perform well relative to other students | · |  |  |
| I am striving to do well compared to other students | · |  |  |
| My goal is to perform better than the other students | · |  |  |

Performance-Avoidance Goal Subscale

|  | Goal | | Non-goal |
| --- | --- | --- | --- |
| Item | Standard | Reason |  |
| My aim is to avoid doing worse than other students | · |  |  |
| I am striving to avoid performing worse than others | · |  |  |
| My goal is to avoid performing poorly compared to others | · |  |  |

Mastery-Avoidance Goal Subscale

|  | Goal | | Non-goal |
| --- | --- | --- | --- |
| Item | Standard | Reason |  |
| My aim is to avoid learning less than I possibly could | · |  |  |
| I am striving to avoid an incomplete understanding of the course material | · |  |  |
| My goal is to avoid learning less than it is possible to learn | · |  |  |

**The Achievement Goal Questionnaire for Sports (AGQ-S) developed by Conroy et al. [46]**

Mastery-Approach Goal Subscale

|  | Goal | | Non-goal |
| --- | --- | --- | --- |
| Item | Standard | Reason |  |
| It is important to me to perform as well as I possibly can | · |  |  |
| I want to perform as well as it is possible for me to perform | · |  |  |
| It is important for me to master all aspects of my performance | · |  |  |

Performance-Approach Goal Subscale

|  | Goal | | Non-goal |
| --- | --- | --- | --- |
| Item | Standard | Reason |  |
| It is important to me to do well compared to others | · |  |  |
| It is important for me to perform better than others | · |  |  |
| My goal is to do better than most other performers | · |  |  |

Performance-Avoidance Goal Subscale

|  | Goal | | Non-goal |
| --- | --- | --- | --- |
| Item | Standard | Reason |  |
| I just want to avoid performing worse than others | · |  |  |
| My goal is to avoid performing worse than everyone else | · |  |  |
| It is important for me to avoid being one of the worst performers in the group | · |  |  |

Mastery-Avoidance Goal Subscale

|  | Goal | | Non-goal |
| --- | --- | --- | --- |
| Item | Standard | Reason |  |
| I worry that I may not perform as well as I possibly can | · |  | · |
| Sometimes I’m afraid that I may not perform as well as I’d like | · |  | · |
| I’m often concerned that I may not perform as well as I can perform | · |  | · |

**The Task and Ego Orientation in Sport Questionnaire developed by Duda and Nicholls [47]**

Task Goal Subscale

|  | Goal | | Non-goal |
| --- | --- | --- | --- |
| Item | Standard | Reason |  |
| I feel most successful in sport when… |  |  |  |
| I learn a new skill and it makes me want to practice more | · |  | · |
| I learn something that is fun to do | · |  | · |
| I learn a new skill by trying hard | · |  | · |
| I work really hard |  |  | · |
| Something I learn makes me want to go and practice more | · |  | · |
| A skill I learn really feels right | · |  | · |
| I do my very best |  |  | · |

Ego Goal Subscale

|  | Goal | | Non-goal |
| --- | --- | --- | --- |
| Item | Standard | Reason |  |
| I feel most successful when… |  |  |  |
| I’m the only one who can do the skill or play | · |  | · |
| I can do better than my friends | · |  | · |
| The others can’t do as well as me | · |  | · |
| Others mess-up and I don’t | · |  | · |
| I score the most points/goals, etc. | · |  | · |
| I’m the best | · |  | · |

**The Perception of Success Questionnaire developed by Roberts et al. [50]**

Task Goal Subscale

|  | Goal | | Non-goal |
| --- | --- | --- | --- |
| Item | Standard | Reason |  |
| In sport, I feel most successful when… |  |  |  |
| I reach my personal goal |  |  | · |
| I show clear personal improvement | · |  | · |
| I perform to the best of my ability | · |  | · |
| I overcome difficulties |  |  | · |
| I reach a goal |  |  | · |
| I work hard |  |  | · |

Ego Goal Subscale

|  | Goal | | Non-goal |
| --- | --- | --- | --- |
| Item | Standard | Reason |  |
| In sport, I feel most successful when… |  |  |  |
| I show others I am the best | · | · | · |
| I’m the best | · |  | · |
| I am clearly superior | · |  | · |
| I outperform my opponents | · |  | · |
| I beat other people | · |  | · |
| I win | · |  | · |

**Patterns of Adaptive Learning Survey developed by Midgley’s et al. [49]**

Mastery-Approach Goal Subscale

|  | Goal | | Non-goal |
| --- | --- | --- | --- |
| Item | Standard | Reason |  |
| I like school work that I’ll learn from, even if I make a lot of mistakes |  |  | · |
| An important reason why I do my school work is because I like to learn new things | · | · |  |
| I like school work best when it really makes me think |  | · |  |
| An important reason why I do my work in school is because I want to get better at it | · | · |  |
| I do my school work because I’m interested in it |  |  | · |
| An important reason I do my school work is because I enjoy it |  |  | · |

Performance-Approach Goal Subscale

|  | Goal | | Non-goal |
| --- | --- | --- | --- |
| Item | Standard | Reason |  |
| I would feel really good if I were the only one who could answer the teachers’ questions in class | · | · | · |
| It’s important to me that the other students in my classes think that I am good at my work |  |  | · |
| I want to do better than other students in my classes | · |  |  |
| I would feel successful in school if I did better than most of the other students | · | · | · |
| I’d like to show my teachers that I’m smarter than the other students in my classes |  |  | · |
| Doing better than other students in school is important to me | · |  |  |

Performance-Avoidance Goal Subscale

|  | Goal | | Non-goal |
| --- | --- | --- | --- |
| Item | Standard | Reason |  |
| It’s very important to me that I don’t look stupid in my classes |  | · |  |
| An important reason I do my school work is so that I don’t embarrass myself |  | · |  |
| The reason I do my school work is so my teachers don’t think I know less than others |  | · |  |
| The reason I do my work is so others won’t think I’m dumb |  | · |  |
| One reason I would not participate in class is to avoid looking stupid |  | · |  |
| One of my main goals is to avoid looking like I can’t do my work |  | · |  |

**The trichotomous Work Domain Goal Orientation Instrument developed by VandeWalle [15]**

Learning Goal Subscale

|  | Goal | | Non-goal |
| --- | --- | --- | --- |
| Item | Standard | Reason |  |
| I am willing to select a challenging work assignment that I can learn a lot from | · |  |  |
| I often look for opportunities to develop new skills and knowledge | · |  |  |
| I enjoy challenging and difficult tasks at work where I’ll learn new skills | · |  | · |
| For me, development of my work ability is important enough to take risks | · |  | · |
| I prefer to work in situations that require a high level of ability and talent |  |  | · |

Performance Prove Goal Subscale

|  | Goal | | Non-goal |
| --- | --- | --- | --- |
| Item | Standard | Reason |  |
| I’m concerned with showing that I can perform better than my coworkers | · | · | · |
| I try to figure out what it takes to prove my ability to others at work | · |  |  |
| I enjoy it when others at work are aware of how well I am doing |  | · | · |
| I prefer to work on projects where I can prove my ability to others |  | · |  |

Performance Avoid Goal Subscale

|  | Goal | | Non-goal |
| --- | --- | --- | --- |
| Item | Standard | Reason |  |
| I would avoid taking on a new task if there was a chance that I would appear rather incompetent to others | · | · |  |
| Avoiding a show of low ability is more important to me than learning a new skill |  | · | · |
| I’m concerned about taking on a task at work if my performance would reveal that I had low ability |  | · | · |
| I prefer to avoid situations at work where I might perform poorly |  |  | · |

**Goal orientation scale developed by Button et al. [45]**

Learning Goal Subscale

|  | Goal | | Non-goal |
| --- | --- | --- | --- |
| Item | Standard | Reason |  |
| The opportunity to do challenging work is important to me |  | · |  |
| When I fail to complete a difficult task, I plan to try harder the next time I work on it |  |  | · |
| I prefer to work on tasks that force me to learn new things | · | · |  |
| The opportunity to learn new things is important to me | · | · |  |
| I do my best when I’m working on a fairly difficult task |  |  | · |
| I try hard to improve on my past performance | · |  |  |
| The opportunity to extend the range of my abilities is important to me | · | · |  |
| When I have difficulty solving a problem, I enjoy trying different approaches to see which one will work |  |  | · |

Performance Goal Subscale

|  | Goal | | Non-goal |
| --- | --- | --- | --- |
| Item | Standard | Reason |  |
| I prefer to do things that I can do well rather than things that I do poorly |  |  | · |
| I’m happiest at work when I perform tasks on which I know that I won’t make any errors | · | · | · |
| The things I enjoy the most are the things I do the best |  |  | · |
| The opinions others have about how well I can do certain things are important to me |  |  | · |
| I feel smart when I do something without making any mistakes | · | · | · |
| I like to be fairly confident that I can successfully perform a task before I attempt it |  |  | · |
| I like to work on tasks that I have done well on in the past |  |  | · |
| I feel smart when I can do something better than most other people | · | · | · |
