# Supplementary material for: A Meta-Analysis of Self-Reported Achievement Goals and Nonself-Report Performance across Three Achievement Domains (Work, Sports, and Education)
Source: PLoS One. 2014 Apr 3;9(4):e93594. doi: 10.1371/journal.pone.0093594 (PMC3974764; doi:10.1371/journal.pone.0093594)
Supplement: Appendix S2 — (DOCX) [file pone.0093594.s002.docx]

**Appendix S2**. Search strategy: Web of Science (timespan 1979 – January 1^st^, 2014)

1. achievement goal/
2. goal orientation/
3. mastery goal/
4. mastery approach goal/
5. mastery-approach goal/
6. approach goal/
7. performance goal/
8. performance approach goal/
9. performance-approach goal/
10. avoidance goal/
11. performance avoidance goal/
12. performance-avoidance goal/
13. mastery avoidance goal/
14. mastery-avoidance goal/
15. learning goal/
16. learning goal orientation/
17. task goal/
18. task goal orientation/
19. prove goal/
20. prove goal orientation/
21. performance prove goal/
22. performance prove goal orientation/
23. ego goal/
24. ego goal orientation/
25. ability goal/
26. performance/
27. performance attainment/
28. 3 or 4 or 5
29. 6 or 7 or 8 or 9
30. 10 or 11 or 12
31. 10 or 13 or 14
32. 15 or 16
33. 17 or 18
34. 19 or 20
35. 21 or 22
36. 23 or 24
37. 26 or 27
38. 1 and 37
39. 2 and 37
40. 28 and 37
41. 29 and 37
42. 30 and 37
43. 31 and 37
44. 32 and 37
45. 33 and 37
46. 34 and 37
47. 35 and 37
48. 36 and 37
49. 25 and 37
