# Supplementary material for: A Meta-Analysis of Self-Reported Achievement Goals and Nonself-Report Performance across Three Achievement Domains (Work, Sports, and Education)
Source: PLoS One. 2014 Apr 3;9(4):e93594. doi: 10.1371/journal.pone.0093594 (PMC3974764; doi:10.1371/journal.pone.0093594)
Supplement: Appendix S5 — (DOCX) [file pone.0093594.s005.docx]

**Appendix S5**

Proposed items for a standards-based achievement goal questionnaire for the *education domain at a broad, general level* (i.e., one’s studies). The items are based on the scales developed by Elliot et al. [85].

**Instructions**: The following statements represent *types of goals* that you may or may not have *in your studies*. Indicate how true each sentence is for you.

All of your responses will be kept anonymous and confidential. There is no right or wrong answer, so *please be open and honest*.

| 1 | 2 | 3 | 4 | 5 | | | 6 | | | | 7 | | |
| --- | --- | --- | --- | --- | --- | --- | --- | --- | --- | --- | --- | --- | --- |
| Not at all true of me | Rarely true of me | Somewhat true of me | Moderately true of me | Reasonably true of me | | | Very true of me | | | | Extremely true of me | | |
| **In my studies, my goal is …** | | | | | | | | | | | | | |
| **[Task-approach goal items]** | | | | | | | | | | | | | |
| 1. .… to get a lot of questions right on the exams. | | | | | 1 | 2 | | 3 | 4 | 5 | | 6 | 7 |
| 2. …. to know the right answers to the questions on the exams. | | | | | 1 | 2 | | 3 | 4 | 5 | | 6 | 7 |
| 3. .… to answer a lot of questions correctly on the exams. | | | | | 1 | 2 | | 3 | 4 | 5 | | 6 | 7 |
| **[Task-avoidance goal items]** | | | | | | | | | | | | | |
| 4. …. to avoid incorrect answers on the exams. | | | | | 1 | 2 | | 3 | 4 | 5 | | 6 | 7 |
| 5. .… to avoid getting a lot of questions wrong on the exams. | | | | | 1 | 2 | | 3 | 4 | 5 | | 6 | 7 |
| 6. .… to avoid missing a lot of questions on the exams. | | | | | 1 | 2 | | 3 | 4 | 5 | | 6 | 7 |
| **[Self-approach goal items]** | | | | | | | | | | | | | |
| 7. …. to perform better on the exams than I have done in the past on these types of exams. | | | | | 1 | 2 | | 3 | 4 | 5 | | 6 | 7 |
| 8. …. to do well on the exams relative to how well I have done in the past on such exams. | | | | | 1 | 2 | | 3 | 4 | 5 | | 6 | 7 |
| 9. …. to do better on the exams than I typically do in this type of situation. | | | | | 1 | 2 | | 3 | 4 | 5 | | 6 | 7 |
| **[Self-avoidance goal items]** | | | | | | | | | | | | | |
| 10. …. to avoid doing worse on the exams than I normally do on these types of exams. | | | | | 1 | 2 | | 3 | 4 | 5 | | 6 | 7 |
| 11. …. to avoid performing poorly on the exams compared to my typical level of performance. | | | | | 1 | 2 | | 3 | 4 | 5 | | 6 | 7 |
| 12. …. to avoid doing worse on the exams than I have done on prior exams of this type. | | | | | 1 | 2 | | 3 | 4 | 5 | | 6 | 7 |
| **[Other-approach goal items]** | | | | | | | | | | | | | |
| 13. …. to outperform others students on the exams. | | | | | 1 | 2 | | 3 | 4 | 5 | | 6 | 7 |
| 14. …. to do well compared to others on the exams. | | | | | 1 | 2 | | 3 | 4 | 5 | | 6 | 7 |
| 15. …. to do better than others on the exams. | | | | | 1 | 2 | | 3 | 4 | 5 | | 6 | 7 |
| **[Other-avoidance goal items]** | | | | | | | | | | | | | |
| 16. …. to avoid performing worse than others on the exams. | | | | | 1 | 2 | | 3 | 4 | 5 | | 6 | 7 |
| 17. …. to avoid doing poorly in comparison to others on the exams. | | | | | 1 | 2 | | 3 | 4 | 5 | | 6 | 7 |
| 18. …. to avoid performing poorly relative to my fellow students on the exams. | | | | | 1 | 2 | | 3 | 4 | 5 | | 6 | 7 |

Proposed items for a standards-based achievement goal questionnaire for the *work domain* *at a broad, general level* (i.e., one’s work). The items are based on the scales developed by Elliot et al. [85].

**Instructions**: The following statements represent *types of goals* that you may or may not have *in your work*. Indicate how true each sentence is for you.

All of your responses will be kept anonymous and confidential. There is no right or wrong answer, so *please be open and honest*.

| 1 | 2 | 3 | 4 | 5 | | | 6 | | | | 7 | | |
| --- | --- | --- | --- | --- | --- | --- | --- | --- | --- | --- | --- | --- | --- |
| Not at all true of me | Rarely true of me | Somewhat true of me | Moderately true of me | Reasonably true of me | | | Very true of me | | | | Extremely true of me | | |
| **In my work, my goal is …** | | | | | | | | | | | | | |
| **[Task-approach goal items]** | | | | | | | | | | | | | |
| 1. .… to get a lot of things right. | | | | | 1 | 2 | | 3 | 4 | 5 | | 6 | 7 |
| 2. …. to know the right answers to relevant questions. | | | | | 1 | 2 | | 3 | 4 | 5 | | 6 | 7 |
| 3. .… to do a lot of things correctly. | | | | | 1 | 2 | | 3 | 4 | 5 | | 6 | 7 |
| **[Task-avoidance goal items]** | | | | | | | | | | | | | |
| 4. …. to avoid incorrect answers or solutions. | | | | | 1 | 2 | | 3 | 4 | 5 | | 6 | 7 |
| 5. .… to avoid getting a lot of things wrong. | | | | | 1 | 2 | | 3 | 4 | 5 | | 6 | 7 |
| 6. .… to avoid missing opportunities. | | | | | 1 | 2 | | 3 | 4 | 5 | | 6 | 7 |
| **[Self-approach goal items]** | | | | | | | | | | | | | |
| 7. …. to perform better than I have done in the past in my work. | | | | | 1 | 2 | | 3 | 4 | 5 | | 6 | 7 |
| 8. …. to do well relative to how well I have done in the past in my work. | | | | | 1 | 2 | | 3 | 4 | 5 | | 6 | 7 |
| 9. …. to improve. | | | | | 1 | 2 | | 3 | 4 | 5 | | 6 | 7 |
| **[Self-avoidance goal items]** | | | | | | | | | | | | | |
| 10. …. to avoid doing worse than I have done in the past in my work. | | | | | 1 | 2 | | 3 | 4 | 5 | | 6 | 7 |
| 11. …. to avoid performance decline. | | | | | 1 | 2 | | 3 | 4 | 5 | | 6 | 7 |
| 12. …. to avoid doing worse than I have done previously in my work. | | | | | 1 | 2 | | 3 | 4 | 5 | | 6 | 7 |
| **[Other-approach goal items]** | | | | | | | | | | | | | |
| 13. …. to outperform others. | | | | | 1 | 2 | | 3 | 4 | 5 | | 6 | 7 |
| 14. …. to do well compared to others. | | | | | 1 | 2 | | 3 | 4 | 5 | | 6 | 7 |
| 15. …. to do better than others. | | | | | 1 | 2 | | 3 | 4 | 5 | | 6 | 7 |
| **[Other-avoidance goal items]** | | | | | | | | | | | | | |
| 16. …. to avoid performing worse than others. | | | | | 1 | 2 | | 3 | 4 | 5 | | 6 | 7 |
| 17. …. to avoid doing poorly in comparison to others. | | | | | 1 | 2 | | 3 | 4 | 5 | | 6 | 7 |
| 18. …. to avoid performing poorly relative to my colleagues. | | | | | 1 | 2 | | 3 | 4 | 5 | | 6 | 7 |

Proposed items for a standards-based achievement goal questionnaire for the *sports domain* *at a broad, general level* (i.e., one’s sports). The items are based on the scales developed by Elliot et al. [85].

**Instructions**: The following statements represent *types of goals* that you may or may not have *in your sports*. Indicate how true each sentence is for you.

All of your responses will be kept anonymous and confidential. There is no right or wrong answer, so *please be open and honest*.

| 1 | 2 | 3 | 4 | 5 | | | 6 | | | | 7 | | |
| --- | --- | --- | --- | --- | --- | --- | --- | --- | --- | --- | --- | --- | --- |
| Not at all true of me | Rarely true of me | Somewhat true of me | Moderately true of me | Reasonably true of me | | | Very true of me | | | | Extremely true of me | | |
| **In my sports, my goal is …** | | | | | | | | | | | | | |
| **[Task-approach goal items]** | | | | | | | | | | | | | |
| 1. .… to get a lot of things right. | | | | | 1 | 2 | | 3 | 4 | 5 | | 6 | 7 |
| 2. …. to do the right things. | | | | | 1 | 2 | | 3 | 4 | 5 | | 6 | 7 |
| 3. .… to do a lot of things correctly. | | | | | 1 | 2 | | 3 | 4 | 5 | | 6 | 7 |
| **[Task-avoidance goal items]** | | | | | | | | | | | | | |
| 4. …. to avoid doing the wrong things. | | | | | 1 | 2 | | 3 | 4 | 5 | | 6 | 7 |
| 5. .… to avoid getting a lot of things wrong. | | | | | 1 | 2 | | 3 | 4 | 5 | | 6 | 7 |
| 6. .… to avoid missing opportunities. | | | | | 1 | 2 | | 3 | 4 | 5 | | 6 | 7 |
| **[Self-approach goal items]** | | | | | | | | | | | | | |
| 7. …. to perform better than I have done in the past in my sports. | | | | | 1 | 2 | | 3 | 4 | 5 | | 6 | 7 |
| 8. …. to do well relative to how well I have done in the past in my sports. | | | | | 1 | 2 | | 3 | 4 | 5 | | 6 | 7 |
| 9. …. to improve. | | | | | 1 | 2 | | 3 | 4 | 5 | | 6 | 7 |
| **[Self-avoidance goal items]** | | | | | | | | | | | | | |
| 10. …. to avoid doing worse than I have done in the past in my sports. | | | | | 1 | 2 | | 3 | 4 | 5 | | 6 | 7 |
| 11. …. to avoid performance decline. | | | | | 1 | 2 | | 3 | 4 | 5 | | 6 | 7 |
| 12. …. to avoid doing worse than I have done previously in my sports. | | | | | 1 | 2 | | 3 | 4 | 5 | | 6 | 7 |
| **[Other-approach goal items]** | | | | | | | | | | | | | |
| 13. …. to outperform others. | | | | | 1 | 2 | | 3 | 4 | 5 | | 6 | 7 |
| 14. …. to do well compared to others. | | | | | 1 | 2 | | 3 | 4 | 5 | | 6 | 7 |
| 15. …. to do better than others. | | | | | 1 | 2 | | 3 | 4 | 5 | | 6 | 7 |
| **[Other-avoidance goal items]** | | | | | | | | | | | | | |
| 16. …. to avoid performing worse than others. | | | | | 1 | 2 | | 3 | 4 | 5 | | 6 | 7 |
| 17. …. to avoid doing poorly in comparison to others. | | | | | 1 | 2 | | 3 | 4 | 5 | | 6 | 7 |
| 18. …. to avoid performing poorly relative to my fellow athletes. | | | | | 1 | 2 | | 3 | 4 | 5 | | 6 | 7 |
