# Supplementary material for: A Meta-Analysis of Self-Reported Achievement Goals and Nonself-Report Performance across Three Achievement Domains (Work, Sports, and Education)
Source: PLoS One. 2014 Apr 3;9(4):e93594. doi: 10.1371/journal.pone.0093594 (PMC3974764; doi:10.1371/journal.pone.0093594)
Supplement: Protocol S1 — PRISMA 2009 Flow Diagram Meta-Analysis. (DOC) [file pone.0093594.s007.doc]

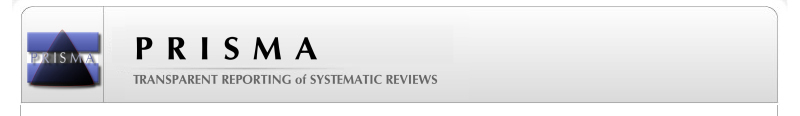
**PRISMA 2009 Flow Diagram**

**Screening**

**Included**

**Eligibility**

**Identification**

Records identified through database searching
(n = 1,477)

Additional records identified through other sources
(n = 12)

Records after duplicates removed
(n = 471)

Records screened
(n = 471)

Records excluded
(n = 370)

Full-text articles assessed for eligibility
(n = 101)

Full-text articles excluded, with reasons
(n = 3)

See eligibility criterion 3 in the manuscript

Studies included in qualitative synthesis
(n = 98)

Studies included in quantitative synthesis (meta-analysis)
(n = 98)
